# The Association between *PARP1* and *LIG3* Expression Levels and Chromosomal Translocations in Acute Myeloid Leukemia Patients

**DOI:** 10.22074/cellj.2018.5210

**Published:** 2018-03-18

**Authors:** Hossein Pashaiefar, Marjan Yaghmaie, Javad Tavakkoly-Bazzaz, Seyed Hamidollah Ghaffari, Kamran Alimoghaddam, Izadi Pantea, Ardeshir Ghavamzadeh

**Affiliations:** 1 Department of Medical Genetics, School of Medicine, Tehran University of Medical Sciences, Tehran, Iran; 2 Hematology, Oncology and Stem Cell Transplantation Research Center, Tehran University of Medical Sciences, Tehran, Iran

**Keywords:** Acute Myeloid Leukemia, Chromosomal Translocation, *LIG3*, *PARP1*

## Abstract

**Objective:**

Chromosomal translocations are among the most common mutational events in cancer development, especially in hematologic malignancies. However, the precise molecular mechanism of these events is still not clear. It has been recently shown that alternative non-homologous end-joining (alt-NHEJ), a newly described pathway for double-stranded DNA break repair, mediates the formation of chromosomal translocations. Here, we examined the expression levels of the main components of alt-NHEJ (*PARP1* and *LIG3*) in acute myeloid leukemia (AML) patients and assessed their potential correlation with the formation of chromosomal translocations.

**Materials and Methods:**

This experimental study used reverse transcription-quantitative polymerase chain reaction (RT- qPCR) to quantify the expression levels of *PARP1* and *LIG3* at the transcript level in AML patients (n=78) and healthy individuals (n=19).

**Results:**

*PARP1* was the only gene overexpressed in the AML group when compared with healthy individuals (P=0.0004), especially in the poor prognosis sub-group. Both genes were, however, found to be up-regulated in AML patients with chromosomal translocations (P=0.04 and 0.0004 respectively). Moreover, patients with one isolated translocation showed an over-expression of only *LIG3* (P=0.005), whereas those with two or more translocations over-expressed both *LIG3* (P=0.002) and *PARP1* (P=0.02).

**Conclusion:**

The significant correlations observed between *PARP1* and *LIG3* expression and the rate of chromosomal translocations in AML patients provides a molecular context for further studies to investigate the causality of this association.

## Introduction

One of the major chromosomal aberrations identified in cancer tissues, particularly in hematological malignancies, is balanced chromosomal translocation, which is thought to play a central role in initiating malignancy. In most cases, these chromosomal abnormalities substantially impact prognosis (1-3). Approximately 25- 30% of adults with de novo acute myeloid leukemia (AML) harbor balanced chromosomal rearrangements (4) which are associated with clinical features and treatment outcome (3). Furthermore, various studies have reported karyotype abnormalities in the form of balanced chromosomal translocations at relapse phases of AML patients (5- 7). It therefore seems that the occurrence of balanced chromosomal translocations contributes to refractoriness against anti-leukemic therapies (7). 

Recent findings have elucidated how balanced translocations are implicated in leukemia development, however, the question of how such translocations are generated in the first place has remained to be fully resolved. It is experimentally clear that these translocations mostly occur due to DNA double-strand breaks (DSBs) in distinct chromosomes (8). DSBs in DNA normally occur by endogenous processes, including DNA replication, DNA repair and rearrangements of immunoglobulin receptor genes. They may also occur through exogenous sources such as exposure to radiation and certain chemicals (9). 

To maintain genomic integrity and protect cells from adverse consequences of these lesions, such as oncogenic transformation following DSB, two major repair pathways are induced, namely homologous recombination (HR), where sequence integrity is preserved, and non- homologous end-joining (NHEJ), which results in small- scale mutations (10). 

Data derived from cloning and sequencing junctions of chromosomal translocations in patients with leukemia have revealed that the breakpoints showed no consistent homologous sequences and often strongly showed repair signatures of NHEJ including small deletions and duplications, non-template insertions and micro- homologies. These observations indicate that these translocations predominantly arise by NHEJ and HR are not thought to play a significant role in chromosomal translocations (1, 2, 11). The NHEJ has two mechanistically distinct pathways, namely the classical (cNHEJ) and the alternative (alt-NHEJ) pathways (10, 12). 

Recent studies have argued against the previous paradigm that NHEJ mediates translocations by demonstrating that chromosomal translocations are more common when cNHEJ components such as KU70 and DNA ligase IV are missing, suggesting that these cNHEJ components repress chromosomal translocations (13, 14). However, several lines of evidence have shown that alt-NHEJ components such as DNA ligase 3 (LIG3), CtIP and poly ADP-ribose polymerase 1 (PARP1) are required for the formation of chromosomal translocations (15-18). 

Since PARP1 and LIG3 are the main components of alt-NHEJ, we aimed to examine the potential role of *PARP1* and *LIG3* in the accelerated rate of chromosomal translocation formation in AML patients. To investigate this possibility, we quantified their expression levels at the transcript level in subsets of AML patients. Our findings suggest that these molecular markers may be used as potential therapeutic targets for AML therapy. 

## Materials and Methods

Bone marrow (BM) specimens were collected from AML patients (n=78) admitted to the Hematology, Oncology and Stem Cell Transplantation Research Center, Shariati Hospital, Tehran, Iran. All patients gave informed written consent for sample collection. This experimental study was approved by the Ethics Committee of Tehran University of Medical Sciences (IR.TUMS.REC.1395.2697). Non-M3 AML patients received standard induction chemotherapy consisting of 1 or 2 courses of daunorubicin (45 mg/m^2^ daily for 3 days) combined with cytarabine (100 mg/m^2^) for a 7-day continuous intravenous infusion. AML-M3 patients received ATRA plus arsenic trioxide for several cycles. Moreover, peripheral blood samples from healthy individuals (n=19) were analyzed as the control group. 

### Specimen collection

Bone marrow samples were collected in EDTA containing tubes. The mononuclear cells (MNCs) were isolated within two hours of bone marrow sample collection using Ficoll-Paque (GE Healthcare, Waukesha, WI, USA) density-gradient centrifugation according to the manufacturer’s instructions. After washing twice using phosphate-buffered saline (PBS, pH=7.4, 0.15 M, Gibco, UK), the cell pellets were directly lysed in TriPure Isolation Reagent (Roche, Germany). 

### Cytogenetic analysis 

Fresh bone marrow aspirate samples were directly cultured and harvested following standard cytogenetic methods. G-banding and FISH (if necessary) were undertaken on each bone marrow sample to detect all cytogenetic abnormalities in accordance with an in-house validated protocol. The chromosomal aberrations in this study were described according to the recommendations of the International System of Human Cytogenetic Nomenclature (ISCN 2013) (19). 

### Total RNA extraction and cDNA synthesis 

Total RNA was isolated from bone marrow MNCs using TriPure Isolation Reagent according to the manufacturer’s procedure (TriPure, Roche, Germany). To determine the quantity and quality of RNA samples, absorbance at 260/280 nm wavelengths was measured by using the NanoDrop spectrophotometer. Moreover, 1 µl of each RNA sample was electrophoresed on a 1.5% agarose gel to observe rRNA bands corresponding to the 28S and 18S subunits, and to assess the integrity of RNA samples. The PrimeScript First Strand cDNA Synthesis kit (Takare, Japan) was used to reverse transcribe 1 µg of total RNA into complementary DNA (cDNA). The synthesized cDNA was stored at -20°C for further analysis. 

**Table 1:** Details of primers used for quantitative polymerase chain reaction


Primer	Primer sequencing (5ˊ-3ˊ)	Amplicon length (bp)	Tm (˚C)

PARP1	F: CCAGGATGAAGAGGCAGTGAAG	147	60.68
	R: TTCTGAAGGTCGATCTCATACTCC		59.42
DNALIG3	F: GGAGGCAGATAGACACAGTATAGG	102	59.54
	R: GGCACCCACAGCAACTAATTC		59.80
ABL1	F: GGAATCCAGTATCTCAGACGAAGTG	227	60.73
	R: GAGGGAGCAATGGAGACACG		60.46
KU70	F: TGGGCAAGATGAAGGCTATCG	162	60.20
	R: CTTCAACCTTGGGCAATGTCAG		60.03


### Quantitative real-time polymerase chain reaction

To assess the relative quantity of mRNA transcripts, qPCR was undertaken in a StepOnePlus Real-Time PCR System (Applied Biosystems, USA) by using the SYBR Green assay in duplicate. The cycling conditions were an initial denaturation step at 95°C for 10 minutes, followed by 45 cycles of 95°C for 10 seconds and 60°C (Combined Annealing/Extension) for 30 seconds. Ultimately a melting curve was generated to ensure primer specificity for each target gene. A standard curve was also generated using a serial dilution (5-fold dilutions) of cDNA samples to determine the efficiency of quantitative polymerase chain reactions (qPCR). All reactions were conducted in a final volume of 20 µl comprising 10 µl qPCR Master Mix (Takara), 2 µl (200 ng/µl) of cDNA, 0.5 µl of each primer and 7 µl of ddH2O. Expression levels of all target genes were normalized with ABL1, a housekeeping gene recommended for such analysis by Europe Against Cancer Program, (20). Relative quantification was undertaken with the 2-..Ct method (21). The primers were designed using the publicly available Primer3 software (22). Details of the primers used are shown in Table 1. 

### Statistical analysis 

Mann-Whitney U test was used to compare PARP1 and LIG3 expression levels between the healthy and AML patient groups. This test was also used to compare the two subgroups of AML patients (with and without chromosomal translocations) with respect to the expression levels of *PARP1* and *LIG3*. A P<0.05 was considered statistically significant. All statistical analyses were implemented in the Statistical Package for Social Sciences (SPSS) version 20 (SPSS, Chicago, IL, USA). 

## Results

Based on the FAB classification, the number of patients in each category were 4 (5.1%) M0, 17 (21.8%) M1, 8 (10.3%) M2, 21 (26.9%) M3, 18 (23.1%) M4, 8 (10.3%) M5, 1 (1.3%) M6 and 1 (1.3%) MDS. Flow cytometric analysis demonstrated that 8 to 90 % (mean of 57.4%) of bone marrow mononuclear cells were immature blood cells. The cells were positive for the surface markers specific for AML subgroups including CD2, CD10, CD13, CD14, CD19, CD33, CD34, CD45, CD64, CD117 and HLA DRI3. All AML samples were negative for TdT (terminal deoxynucleotidyl transferase). The control group comprised the healthy individuals aged from 20 to 66 years (median=35), of whom 50 % were male. The patients with AML (39 females and 39 males) were between 14 and 76 years of age (median=39.5 years). The patient diagnosis was based on the revised French-American-British (FAB) classification. The clinic-pathological characteristics of patients are given in Table2. 

### *PARP1* but not *LIG3* was up-regulated in de novo acute myeloid leukemia patients

**Fig.1:**
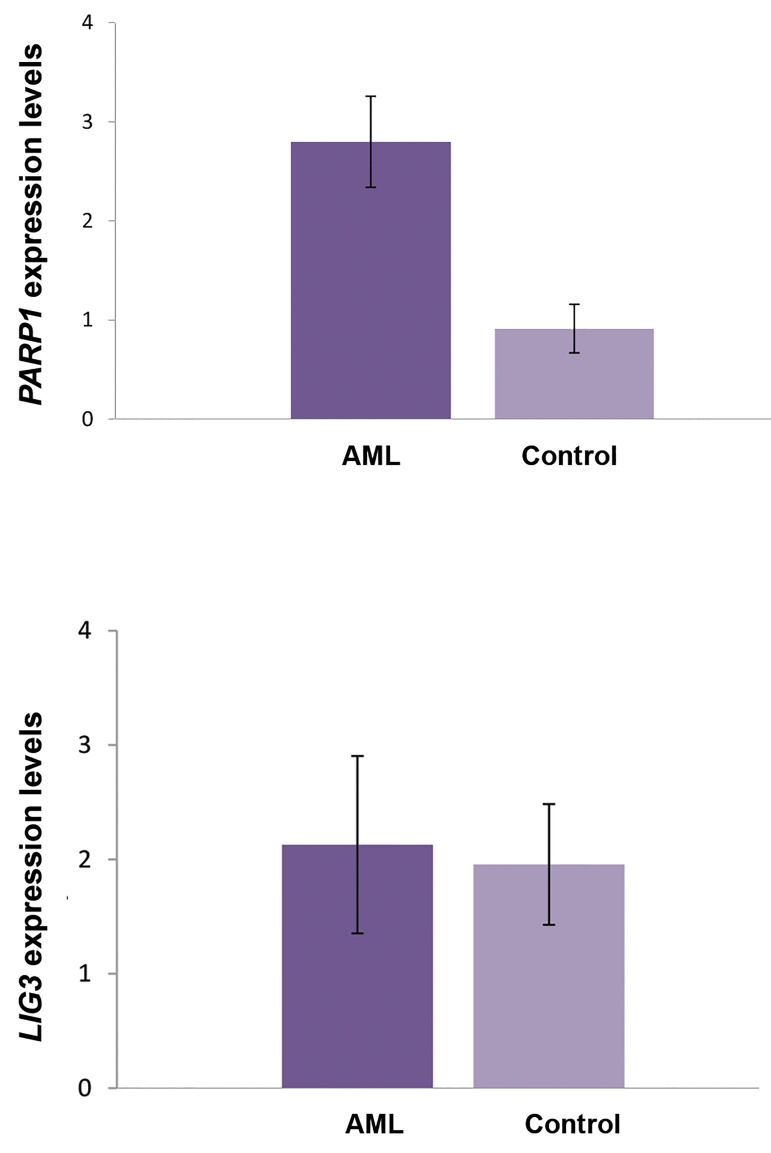
Expression change analysis of *PARP1* and *LIG3* in acute myeloid leukemia (AML) patient and the control groups. While the quantitative polymerase chain reaction (qPCR) results showed a significant up- regulation for PARP1 transcript (P=0.0004), LIG3 did not show a significantly differential expression in AML patients (P=0.08).

By comparing the AML and control groups, PARP1 was significantly differentially expressed (3.09-fold up-regulation, P=0.0004) (Fig .1). The normalized expression levels of *PARP1* in different cytogenetic risk-based subgroups of AML patients are summarized in Table 3. In contrast, no statistically significant difference was observed for *LIG3* expression (P=0.08) in the overall comparison and also between AML subgroup with structural chromosomal aberrations and healthy controls (P=0.67). Interestingly, *PARP1* was also significantly upregulated in the poor prognosis group when compared with the good or intermediate prognosis subgroup (P=0.01). 

**Table 2:** Clinical and genetic characterization of acute myeloid leukemia (AML) patients


Sample type	Number of sample	Chromosomal aberrations	Gene mutations	Median age	Male/Female

Translocation positive AML (BM)	43	t(15;17) (n= 21)	NPM1; (n=4)	38 (17-76)	21/22
		t(8;21) (n= 2)	FLT3 ITD; (n=6)		
		Inv 16 (n=3)			
		t(9;11) (1)			
		t(11;19) (1)			
		2≤translocations (n=15)			
Translocation negative AML (BM)	35	Normal and aneuploid karyotype	NPM1; (n=18)	43 (14-73)	18/17
			FLT3 ITD; (n=17)		
Healthy (PB)	19			35 (20-66)	9/10


**Table 3:** The relative expression levels of PARP1 in different subgroups of acute myeloid leukemia (AML) patients based on cytogenetic risk


Cytogenetic risk	Sample size	Fold-change	P value

**Favorable and Intermediate**	49	2.533	0.005
**Adverse**	29	4.358004	0.0001


### *PARP1* and *LIG3* are up-regulated in patients with chromosomal translocations

**Fig.2:**
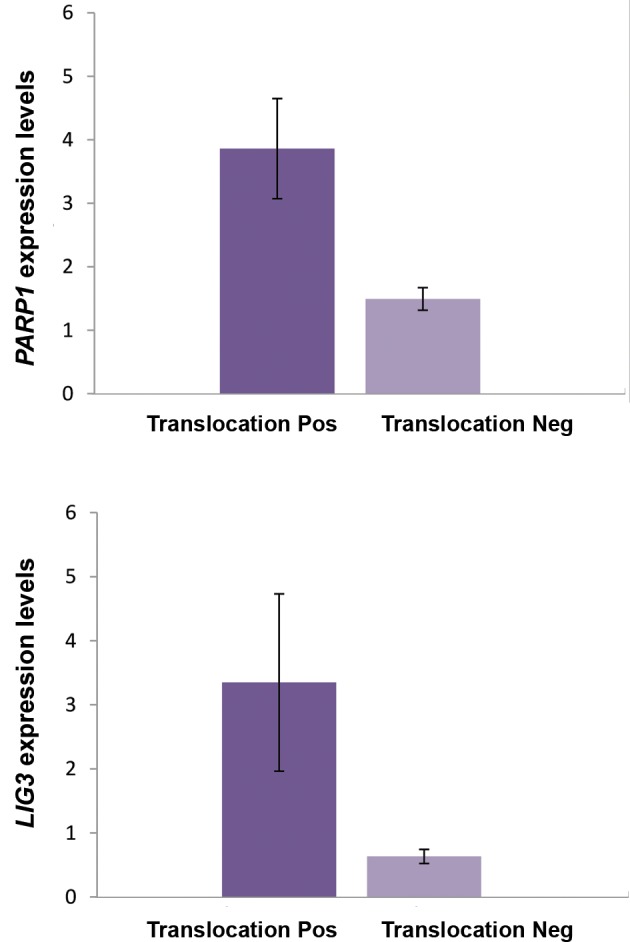
*PARP1* and *LIG3* expression levels in in acute myeloid leukemia (AML) patients with and without chromosomal translocations. LIG3 and PARP1 were significantly up-regulated in AML patients with chromosomal translocations (P=0.04, P=0.0004), respectively).

In order to assess the potential correlation of *PARP1* and *LIG3* expression with the presence of chromosomal translocations, we subgrouped AML patients based on presence of chromosomal translocations and compared the expression levels of *PARP1* and *LIG3* between the two subgroups. *LIG3* and *PARP1* were significantly up-regulated in the subgroup with chromosomal translocations (P=0.04 and P=0.0004, respectively) (Fig .2). We further divided this subgroup into: i. AML patients with one isolated chromosomal translocation and ii. AML patients with two or more chromosomal translocations. The expression levels of *PARP1* and *LIG3* transcripts were compared (Table 4) and consistently, only LIG3 showed significant dysregulation by being 2.23-fold up-regulated (P=0.005). However, when the patients with two or more than two translocations were compared with patients with no translocations, both LIG3 and PARP1 were significantly overexpressed (P=0.002 and P=0.02 respectively).

**Table 4:** Expression levels of PARP1 and LIG3 transcripts in acute myeloid leukemia (AML) patients according to the number of chromosomal translocations observed


	PARP1	P value	LIG3	P value

AML patients with one isolated chromosomal translocation	2.051546	0.2	2.231693	0.005
AML patients with two or more than two chromosomal translocations	2.504776	0.02	3.847066	0.002


### *KU70* expression levels were not altered in acute myeloid leukemia patients with chromosomal translocations 

To examine the involvement of the classical NHEJ pathway in different chromosomal translocation-based subgroups, we analyzed the mRNA expression level of *KU70*, a component of the classical pathway, in bone marrow specimens of AML patients. *KU70* was not significantly dysregulated in AML patients according to absence or presence of chromosomal translocations (P=0.08). 

## Discussion

Wide spectra of hematologic malignancies display one or several balanced chromosomal translocations. In most cases, these aberrations have a major role in diagnosis and treatment of hematological disorders. Although the precise underlying molecular mechanism behind these translocations are yet unknown, recent studies have demonstrated that unlike the components of the cNHEJ pathway, alt-NHEJ factors such as CtIP, DNA ligase III and PARP1 are required for chromosomal translocation formation (15, 16, 18). 

In this study, we hypothesized a putative correlation between *PARP1* and *LIG3* expression levels and formation of chromosomal translocations in AML patients. Given the overexpression of PARP1 and not LIG3 in the overall AML patient group, especially in patients with poor prognosis, suggests a potential role of PARP1 in the development of AML. This result, on its own, is particularly significant since several PARP inhibitors have been well-tolerated in clinical trials of breast and ovarian cancer and may therefore be potential candidates for AML therapy (23).

Our findings are in agreement with those published by Wang et al. (24), by not only showing *PARP-1* up- regulation in AML patients, but also demonstrating that parp-1 inhibition suppresses the proliferation of AML cells, induces apoptosis *in vitro* and improves AML prognosis in mice.

Given the involvement of PARP-1 in a wide variety of cellular functions, including inflammation, gene transcriptional regulation, cell cycle progression, energy metabolism, cell proliferation and cell death (25, 26), PARP-1 is deemed as an oncogene. It has been found that PARP-1 is overexpressed in various types of human cancers including breast cancer (27), prostate cancer (28), hepatocellular carcinoma (29), gastric cancer (30) and nasopharyngeal carcinoma (31). Moreover, its overexpression is inversely correlated with the overall prognosis. It is thought that PARP-1 plays its oncogenic role by mechanistically different pathways. Wang et al., showed that PARP-1 expression was positively correlated with myeloproliferative leukemia virus oncogene (MPL) expression in AML patients (24). In this study, however, we focused on the potential roles of *PARP1* and *LIG3* as main components of alt-NHEJ in generating balanced chromosomal translocations in AML patients and analyzed their expression at the transcript level in AML subgroups based on presence or absence of translocations.

*LIG3* consistently showed significant upregulation at all comparison levels, proposing a contributory role for this gene in the emergence of translocations, nevertheless, the precise mechanism should be further explored. On the other hand, *PARP1* was not always significantly upregulated in all comparisons. For instance, when all patients with at least one translocation are compared, only *LIG3* is overexpressed, however, when patients with two or more translocations are compared with those with an isolated translocation, both genes are significantly up- regulated with *PARP1* showing borderline significance, suggesting that PARP1 is most likely associated with severity of genetic risk.

These data also suggest the probable synergic effect of *LIG3* and *PARP1* on the formation of translocations in patients with AML where simultaneous overexpression of both genes may have an inducing effect on the generation of translocations. These data are supported by recent reports suggesting the physical and functional interactions between LIG3 and PARP1 (32).

To exclude the classical NHEJ pathway in generating chromosomal translocations, we quantified the expression level of *KU70* and observed no evidence for significant dysregulation of this gene in AML patients based on absence or presence of chromosomal translocations, suggesting that formation of chromosomal translocation in AML patients is less likely to be exerted through the cNHEJ pathway.

Our findings are consistent with previous studies analyzing the expression of genes implicated in cNHEJ and altNHEJ in patients with hematologic malignancies. It has been shown that downregulation of Ligase IV (major ligase in the cNHEJ pathway) may potentially induce genetic instability and complex cytogenetic abnormalities in myelodysplastic (MDS) patients (33). Pournazari et al. (34) have also shown a positive association between high PARP-1 expression and a complex karyotype in patients with B-Cell Acute Lymphoblastic Leukemia (B-ALL). Other studies have shown the overexpression of PARP-1 in 61% of adult ALL patients with B-ALL and in ALL children with poor response to treatment (35) suggesting a potential role for PARP-1 in ALL development. 

Finally, many studies have reported karyotype instability and development of chromosomal translocations in patients with relapsed leukemia, especially those affected by AML (5-7). Kern et al. (7), reported that balanced chromosomal translocations were developed in 7 of 20 patients with initially normal karyotypes during relapsing periods. This suggests that balanced chromosomal translocations may occur between diagnosis and the relapse phase and result in refractoriness to anti-leukemic therapy, thus the evaluation of these chromosomal aberrations can be regarded as a marker predicting the relapse phase.

## Conclusion

The findings presented here have clinical significance under three perspectives. First, it is possible that increased *PARP1* expression increases the rate of leukemogenic translocation formation. Accordingly, due to the availability and tolerability of *PARP1* inhibitors, it is possible to reduce the risk of translocation development by using a concurrent treatment with *PARP1* inhibitors as a reasonable therapeutic option to reduce the risk of developing further translocations, hence avoiding the emergence of relapse and therapy resistance in AML patients. Second, our results highlight the importance of *PARP1* and *LIG3* overexpression as different risk markers of translocation formation. 

This association may be used for predicting risk of translocation formation and secondary leukemia in patients undergoing high risk therapy. Thirdly, given the correlation between the expression levels of PARP1 and LIG3 with formation of translocations and the possible occurrence of these chromosomal aberrations between diagnosis and the relapsing stage, we suggest measuring these two markers to predict the translocation formation and possibly to introduce them as novel therapeutic targets to prevent the relapsing phase in AML. However, further investigations are required to fully establish this association.
